# Parametric Investigation into the Shear Strength of Adhesively Bonded Single-Lap Joints

**DOI:** 10.3390/ma15228013

**Published:** 2022-11-13

**Authors:** Quanlong Chen, Bing Du, Xiaodong Zhang, Hai Zhong, Conggang Ning, Huimin Bai, Qian Li, Ruqing Pan, Baocheng Zhou, Hanjie Hu

**Affiliations:** 1 School of Aeronautics, Chongqing Jiaotong University, Chongqing 400074, China; 2 The Green Aerotechnics Research Institute of CQJTU, Chongqing 401120, China; 3 Chongqing Key Laboratory of Nano–Micro Composite Materials and Devices, School of Metallurgy and Materials Engineering, Chongqing University of Science and Technology, Chongqing 401331, China; 4 Innovation Center, Chongqing Polycomp International Corp., Chongqing 401321, China

**Keywords:** composite laminate, single lap joint, adhesively bonding, geometric parameters, failure strength

## Abstract

In this paper, the shear strength of adhesively bonded single-lap joints were experimentally and numerically investigated. Based on the validated simulation, the effects of lap length, adhesive layer thickness, adhesive layer shape, adhesive layer overflow length, and laminate lay-up on the shear strength of adhesively bonded single-lap joints were studied. The load-displacement curves and shear strength under different parameters were compared. It was shown that the shear strength of single-lap joints gradually decreases with the increase of lap length and adhesive layer thickness, which were 53.83% and 16.15%, respectively. Considering the potential condition in fabrication, the adhesive layer shape and adhesive layer overflow length were also investigated. The adhesive with normal and triangle shape owned the comparable shear strength, which was higher than the arc one. The shear strength increased by 19.37% from 18.43 MPa to 22.00 MPa with increasing the adhesive layer overflow length to 50% of lap length. It was beneficial for shear strength to increase the adhesive layer overflow length to 50% of lap length. Among the selected four lay-ups, [0]_16s_ had the highest shear strength, which was nearly 3 times greater than the one of [90]_16s_. In the real process preparation, increasing the number of 0° layers, selecting the appropriate lap length and thickness of the adhesive layer, and controlling the shape and length of the adhesive layer overflow are of great help to improve the tensile shear strength of the single-lap glue joint.

## 1. Introduction

Composite materials are widely used in aerospace, shipbuilding, wind power generation, and other industries because of their high specific strength and specific modulus. Although composite materials have good integrity, the problem of connection between structures is inevitable due to a series of factors such as structural design and manufacturing cost. The main connection methods of composite materials are adhesive connections, mechanical connections, stitching connections, Z-Pin connections, and hybrid connections [1]. Adhesive joint, as a form of connection, is the most widely used type of composite material connection compared to several other types of connections, as it maintains the integrity of the structure, reduces the weight of the joint, and enhances the sealing of the structural joint. There are various types of lap structures for adhesive joint, mainly single-lap [2,3,4], flat-fold-flat type [5], wave type [6], etc. Damage failure occurs when the strength of the adhesive joint itself cannot support the strength of the outside world. According to the location where the damage occurs, the failure form of the joint can be divided into four forms: parent material fracture, cohesive failure, interfacial failure, and mixed failure [7].

Chen et al. [8] studied the experimental shear strength of single-lap joints prepared by three different forming processes, RTM, RTM with the introduction of sewing and adhesive joint under ambient temperature, and hot and humid high-temperature environments, and analyzed the shear damage mechanism of single lap joints under the three preparation methods and the effect of ambient temperature on the shear strength of single-lap joints based on the experimental phenomena. Li [9] prepared a new self-inserting adhesive joint structure and studied the effects of lap length, inflection point, and lap surface on the bearing capacity of the joint. Zhang et al. [10] studied the shear damage test of the adhesive surface of the composite single-lap adhesive structure and obtained the ultimate shear strength of the structure. Wu et al. [11] studied various forms of adhesive joints of composites and analyzed the stresses in the adhesive joints. Liang et al. [12] conducted experimental studies and numerical simulations on adhesive joints of laminates with different lap lengths and found that the failure modes and ultimate loads of adhesive joints are related to the length of the adhesive joint and the thickness of the adhesive parts based on the damage morphology of the joints. The use of the finite element method for parametric study and analysis of composite adhesive joint is more common. The study includes the factors affecting the stresses in the adhesive layer and the strength of the joint, such as the lap length [12], the thickness of the adhesive layer [13,14,15,16], the lay-up angle [17], the design of the joint end [18], etc. It also includes the models for the analysis of the damage of the adhesive layer and the prediction of the joint strength. In particular, finite element methods based on cohesion and continuum damage models have been developed recently to analyze the damage of adhesive layers and the damage of composite interfaces [19,20]. Xu et al. [21] investigated flat-fold-flat (FJF) adhesive joints by ABAQUS software and compared the strength of flat single-lap adhesive joints and found that FJF lap joints have a larger damage tolerance compared to the flat-lap method.

From the above, it can be concluded that many factors, such as lap length, adhesive layer thickness, and laminate lay-up, have influence on the shear strength of the single-lap joint. However, the factors named lap overflow adhesive layer shape and lap overflow length, which can be met in the fabrication process, are investigated relatively less. In this paper, the shear strength of adhesively bonded single-lap joints were experimentally and numerically investigated. Based on the validated simulation, the effects of lap length, adhesive layer thickness, adhesive layer shape, adhesive layer overflow length, and laminate lay-up on the shear strength of adhesively bonded single-lap joints were studied. The load-displacement curves and shear strength under different parameters were compared. Moreover, the interaction between the above factors was studied to select the key factor by the Design of Experiment (DOE) method.

## 2. Methods

### 2.1. Fabrication and Experiment

The laminate material is ZPNPREG^®^2551/150/37 carbon fiber/epoxy prepreg, provided by Shangwei (Shanghai, China) Carbon Fiber Composites Company Limited (SWCFC). The specific material mechanical property parameters are shown in Table 1; the single-lap zone bonding material is LORD^®^320/322 epoxy structural adhesive, and the performance parameters are shown in Table 2; the reinforcing sheet zone bonding material is 3M epoxy structural adhesive.

**Figure 1:**
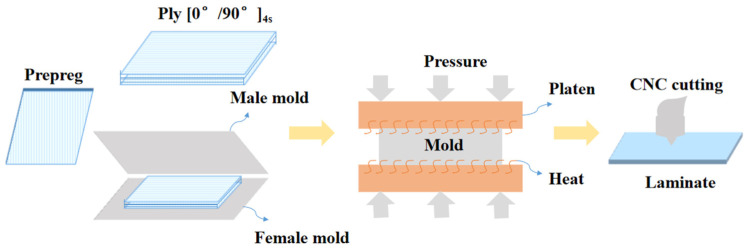
Fabrication of single-lap laminate.

**Figure 2:**
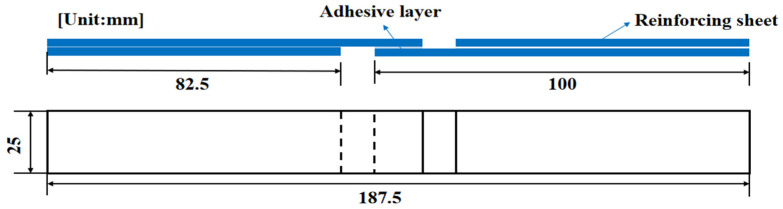
Schematic diagram of single-lap specimen.

$K_{nn}$, $K_{ss}$, and $K_{tt}$ refer to stiffnesses in the normal direction, the first shear direction, and the second shear direction, respectively. $\sigma_{n}$, $\sigma_{s}$, and $\sigma_{t}$ refer to peak values of the nominal stress in the normal direction, the first shear direction, and the second shear direction, respectively. $G_{nn}$, $G_{ss}$, and $G_{tt}$ refer to separation energies in the normal direction, the first shear direction, and the second shear direction, respectively. The laminate lay-up design was [0/90]_4s_; the laminate was prepared by molding process, and the single-lap specimen was prepared by cutting with 4060 CNC engraving machine, as shown in Figure 1. The geometry of the single-lap specimen is 100 × 25 × 2.4 mm, the length of the lap area is 12.5 mm, and the geometry of the reinforced sheet specimen is 82.5 × 25 × 2.5 mm, as shown in Figure 2.

**Figure 3:**
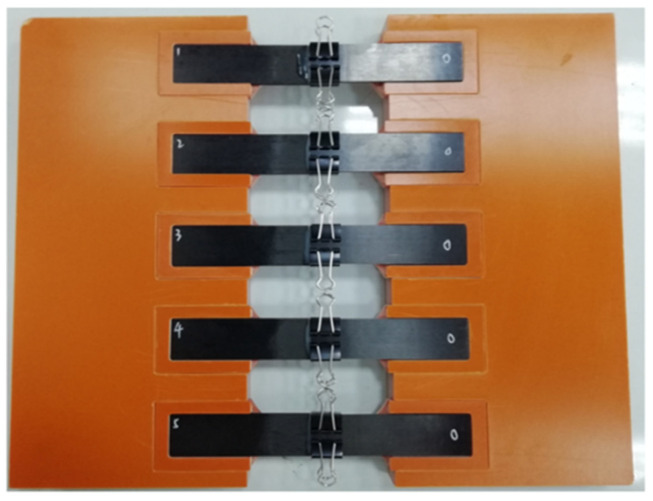
The fabricated single-lap specimen.

The specimens were prepared in two steps, the first for gluing of the lap zone and the second for gluing of the reinforcement piece. Each process contains three parts: surface grinding, application of epoxy structural adhesive, and curing at room temperature (23 ± 5) °C for 24 h. Surface treatment was conducted as in reference [22]: firstly, random sanding with 220-grit sandpaper, followed by alcohol wiping of impurities on the adhesive surface after sanding; application of structural adhesive was completed within 20 min; the fixing pressure was applied using long-tail clamps with a pressure of less than 0.1 MPa. The laminate single-lap adhesive specimens are shown in Figure 3.

**Figure 4:**
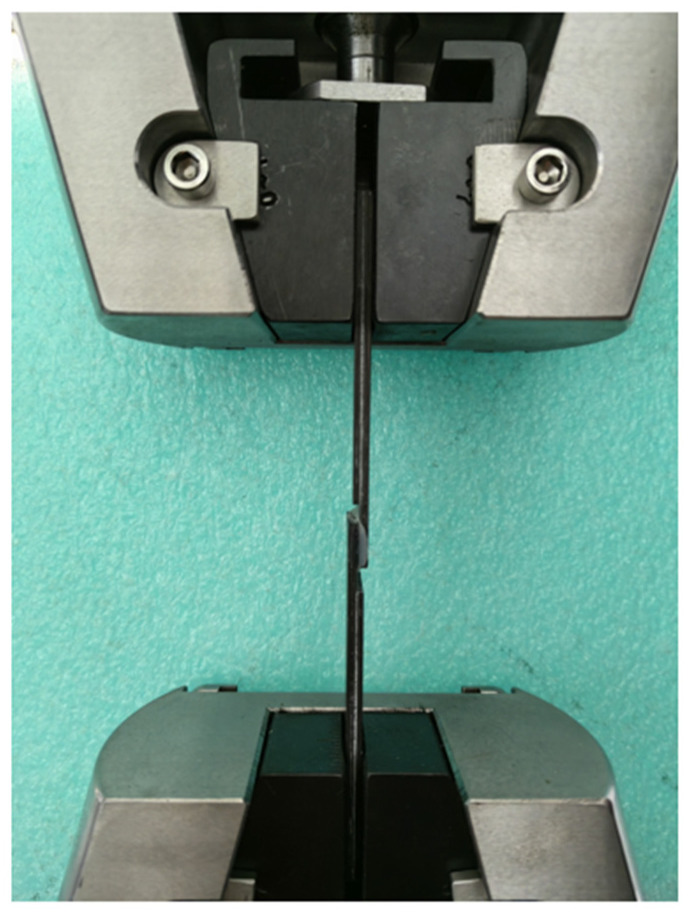
Tensile shear test of single-lap specimen.

The lap shear testing was conducted on a UTM5105X electronic universal testing machine according to GB/T 3334-2016 with the speed of 1.3 mm/min [23,24]. The specimen is fixed as shown in Figure 4. Tensile shear strength of the specimen can be calculated by

(1) $$\tau =F_{m}/\left(B\times L\right)$$

where τ is tensile shear strength in MPa (MPa); $F_{m}$ is test maximum force in Newtons (N); B is bonding area width in millimeters (mm); *L* is bonding area length in millimeters (mm).

### 2.2. Simulation

**Figure 5:**
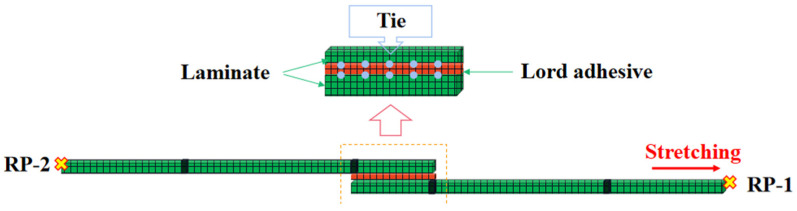
Simulation set-up.

The finite element part uses ABAQUS/Explicit to establish the single-lap tensile shear model. The model includes the establishment of laminate and adhesive layer; the laminate adopts 4-node quadrilateral linear-reduced integral form of shell cells (S4R), which can effectively ensure the accuracy and efficiency of the calculation; the adhesive layer is divided by three-dimensional cohesive cells (COH3D8); and the adhesive layer is meshed by sweeping in the mesh partitioning. The parameters in Table 1 and Table 2 are used for composite and adhesive layer, respectively. The cohesive bilinear model is chosen where the Quads Damage criterion and B-K mixed-mode energy criterion are used. After the convergence test, the mesh size of 1 mm and step time of 0.1 s are chosen. In this paper, the mesh size is chosen to be 1 mm, the laminate is connected to the adhesive layer by face-to-face binding, the displacement is loaded along the *X*-axis by establishing the reference point and coupling constraint to the edge, and the fixed end is completely fixed by establishing the reference point. The end is completely fixed in degrees of freedom, and the specific modeling is shown in Figure 5.

## 3. Results and Discussion

### 3.1. Validation of Simulation Results

**Figure 6:**
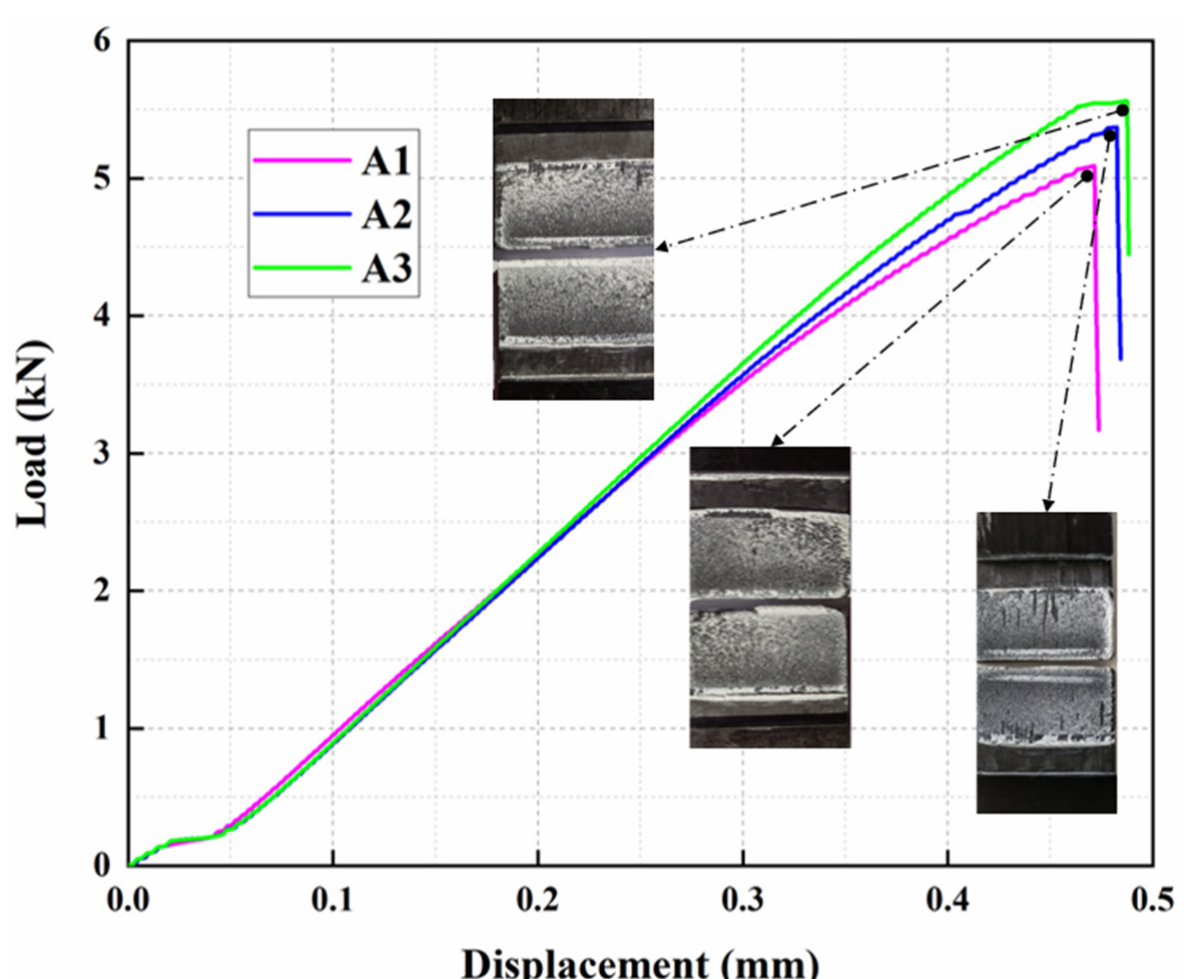
The load-displacement curves of single-lap specimens.

The experimental results of LORD adhesive are shown in Table 3, and the load-displacement curves and specimen cross-sections obtained from the experiments are shown in Figure 6. From the cross-sectional view of the specimens in Figure 6, the specimens all have both structural adhesive and plate interface failure and structural adhesive cohesive failure, which are mixed failure modes, where the failure mode of the adhesive joint is mainly cohesive failure, i.e., the adhesive layer is damaged internally due to insufficient shear strength. The load displacement curve is linear-elastic at the initial tension, and the adhesive layer undergoes linear-elastic deformation. After reaching the peak load, the internal stress of the adhesive layer meets the damage initiation criterion, and local failure starts to occur. Then, the failure area of the adhesive layer expands until the final total failure. The average peak load of the adhesive joint specimen is 5342.07 N, and the average tensile shear strength is 17.00 MPa, with the average maximum displacement of failure at 0.48 mm. As shown in specimen cross-sectional diagram, adhesive cohesive failure happened, indicating that the adhesive layer is damaged internally due to insufficient shear strength.

**Figure 7:**
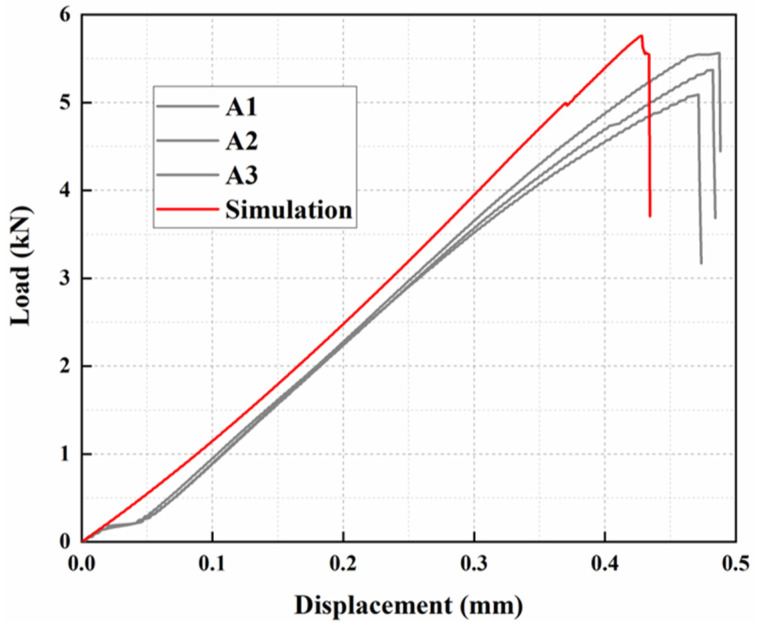
Comparison of experimental and FE simulation results.

The simulated and experimental curves are compared as in Figure 7, from which it is found that there is a difference between the displacement of the simulated curve and the experimentally obtained displacement. The average failure load error between simulation and experiment is 7.83%, which is within the reasonable range and proves the validity of simulation.

### 3.2. Effect of Lap Length

**Figure 8:**
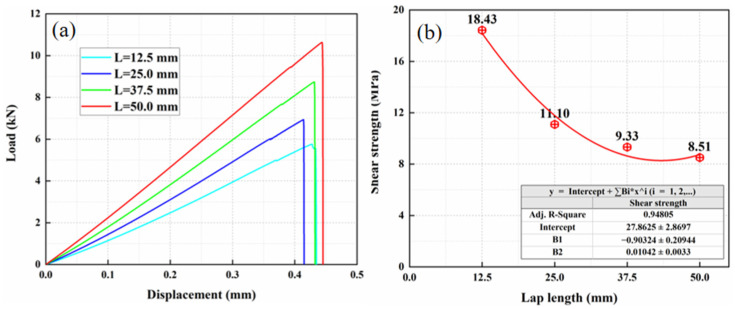
The result of different lap lengths: (**a**) load displacement curves and (**b**) shear strength.

In order to study the effect of lap length on the strength of composite adhesive joint, the lap length *L* was varied with other parameters identical, and four lap lengths were set as 12.5 mm, 25 mm, 37.5 mm, and 50 mm, respectively. From Figure 8, it is found that the shear strength of single-lap joints gradually decreases with the increase in lap length, and this decline is slowing down. Although decreasing the lap length is beneficial for improving the shear strength, special attention should also be paid to choosing this parameter, considering the fabrication problem and requirements of other properties.

### 3.3. Effect of Adhesive Layer Thickness

**Figure 9:**
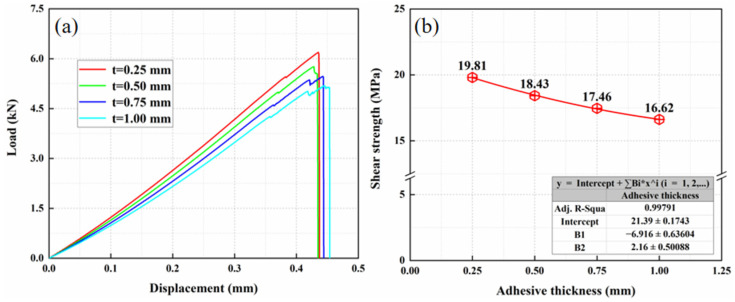
The result of different adhesive layer thickness: (**a**) load displacement curves and (**b**) shear strength.

In order to study the effect of the adhesive layer thickness on the shear strength of the composite adhesive joint, the thickness *t* of the adhesive layer was varied with the other identical parameters, and four adhesive layer thicknesses were set as 0.25 mm, 0.5 mm, 0.75 mm, and 1.00 mm, respectively. The load displacement curves and shear strength of the single-lap specimen with a different adhesive layer thickness is depicted in Figure 9. It is shown that the shear strength of single-lap joints gradually decreases with the increase in adhesive layer thickness. When adhesive thickness increases from 0.25 mm to 1.00 mm, the 16% drop of the shear strength is found, which is from 19.81 MPa to 16.62 MPa, where a similar result has been found in [25]. Qin et al. [26] found that when the adhesive layer thickness increased from 2.0 mm to 5.0 mm, the damage load of the adhesive joint decreased overall.

### 3.4. Effect of Adhesive Layer Shape

**Figure 10:**
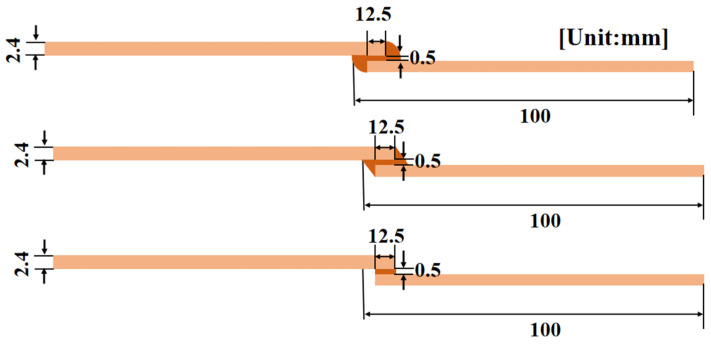
Schematic diagram of different adhesive layer shapes.

**Figure 11:**
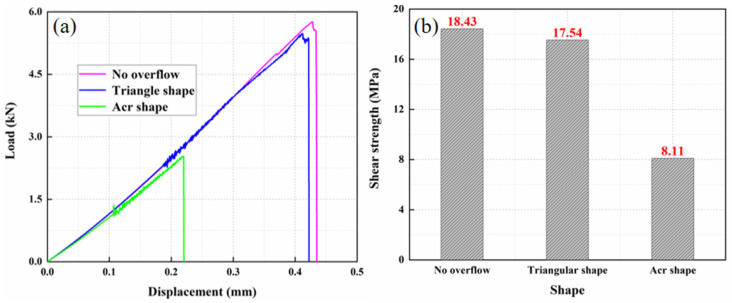
The result of different adhesive layer shapes: (**a**) load displacement curve and (**b**) shear strength.

Considering the potential condition in fabrication, the squeezing of adhesive layer will be found as the forms of contact of laminate end and overflow onto the surface. Investigation on the influence of adhesive layer shape and adhesive layer overflow length were conducted. The lap shape of overflow was changed under the other identical parameters, and three shapes were named circular, triangular, and unoverflowed, and their schematic diagram is shown in Figure 10. In Figure 11, it is shown that the adhesive with the normal and triangle shape owned the comparable shear strength, which was 2 times greater than that of the arc one. Meanwhile, the initial stiffness in load-displacement curves are nearly the same.

### 3.5. Effect of Adhesive Layer Overflow Length

**Figure 12:**
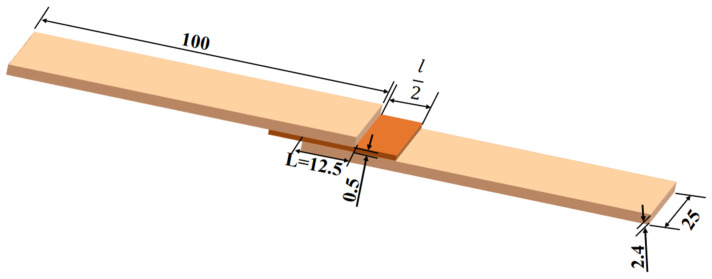
Schematic diagram of different adhesive layer overflow length.

**Figure 13:**
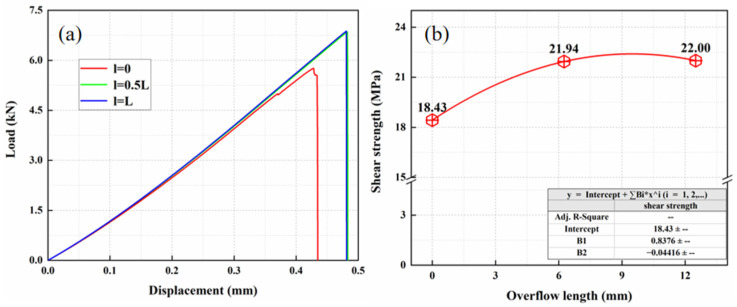
The result of adhesive layer overflow length: (**a**) load displacement curves and (**b**) shear strength.

In order to study the influence of the overflow length of structural adhesive on the strength of composite adhesive joint during lap joint, the overflow length *l* was changed under the premise that other parameters were the same, and three lengths were set as 0, 0.5*L*, *L*. The schematic diagram is shown in Figure 12, the load displacement curves of the adhesive head under three overflow lengths are shown in Figure 13a,b is the shear strength under three overflow lengths. It was beneficial for shear strength to increase the adhesive layer overflow length to 50% of lap length. 

**Figure 14:**
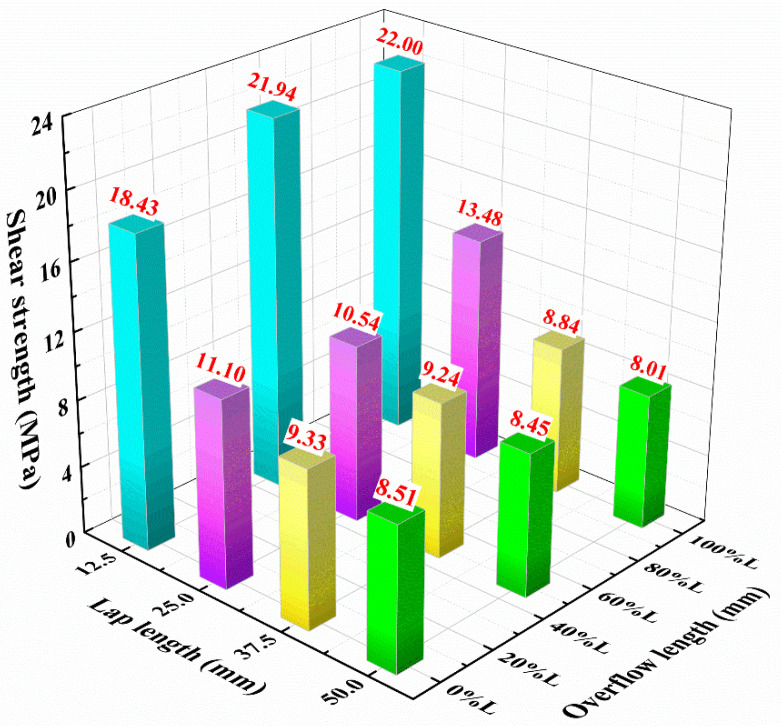
Effect of overflow length on tensile shear strength at different lap lengths.

In the real fabrication process, it is inevitable that the structural adhesive will overflow onto the part of the lap area length. The overflow lengths under each lap length are defined as 0*L*, 0.5*L*, *L*. As shown in Figure 14, tensile shear strength increases with the increase in overflow length when the lap length is 12.5 mm; when the lap length is 25 mm, the tensile shear strength first decreases and then increases; when the lap length is 37.5 mm and 50 mm, the tensile shear strength decreases with the increase in overflow length. Neto et al. [2] used a cohesive zone model to simulate the adhesive layer and established a prediction criterion for joint failure. It was found that the joints with brittle adhesives (AV138) undergo damage in the adhesive when the lap length is in the range of 10–20 mm. The damage of the joints exhibits interlaminar damage of the bonded composite when the lap length is between 30–80 mm. For the results shown in Figure 14, a potential reason is that the distribution of the shear stress is influenced by the overflow length. An increase in the overflow length leads to a decrease in the Mode II toughness of the interface [27,28,29]. As a result, earlier cracking starts and extends at the interface, leading to a reduction in the strength of the joint.

### 3.6. Effect of Laminate Lay-Up

**Figure 15:**
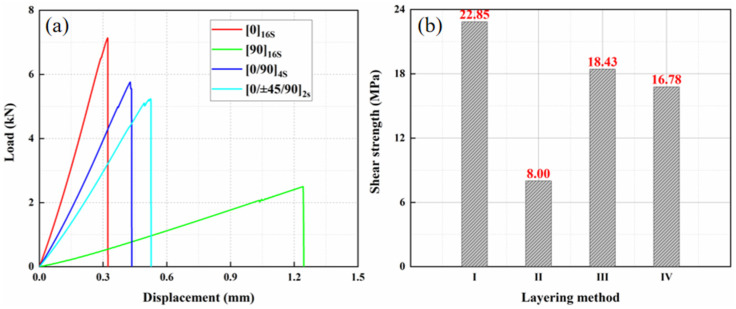
The result of different lay-ups: (**a**) load displacement curves and (**b**) shear strength.

In order to study the influence of the lay-up angle of the laminate on the strength of the composite adhesive joint, four lay-ups were set up with [0]_16s_, [90]_16s_, [0/90]_4s_, and [0/ ± 45/90]_2s_ by changing the lay-up angle while keeping the other parameters identical, and Figure 15a shows the load displacement curves of the adhesive head under the four lay-ups, and Figure 15b shows the shear strength. According to the results, the higher the proportion of 0° lay-up, the greater the shear strength of single-lap. Among the selected four lay-ups, [0]_16s_ had the highest shear strength, which was nearly 3 times greater than the one of [90]_16s_.

It is known from other scholars that the higher the proportion of 0° plies, the higher the single-lap glued tensile shear strength of the laminate [30], and the maximum tensile shear strength under [0]_16s_ plying method is due to the fact that the plying fiber direction of the CFRP composite laminate adjacent to the adhesive layer is 0°, and the laminate can withstand larger tensile loads; the minimum tensile shear strength under [90]_16s_ plying method is due to the fact that the plying fiber direction of the CFRP composite laminate adjacent to the adhesive layer is 90°, and the laminate cannot withstand larger tensile loads.

### 3.7. Multifactor Interaction

Based on the original study of a single factor, an orthogonal experimental table was designed, and the results were obtained by model calculations in Table 4 and analyzed in Table 5 to study the interactive effects of each factor of lap length, adhesive layer thickness, adhesive layer overflow length, and laminate lay-up method on tensile shear strength.

The closer *R*^2^ is to 1, the better the model fits the results. The model variance *R*^2^ = 0.963 indicates that 96% of the experimental data can be explained by this model. The larger the *F* value of the model, the less the *p*-value represents the more significant effect of the correlation coefficient. The 4-factor ANOVA was used to analyze the relationship between the effects of lap length, adhesive layer thickness, lap spillage length, and laminate lay-up on tensile shear strength. From Table 4, the lap length showed a significant effect on the tensile shear strength with *F* = 14.632, *p* = 0.013 < 0.05; the adhesive layer thickness and overflow length did not show significance on the tensile shear strength with *F* = 3.678, *p* = 0.12 > 0.05 and *F* = 2.143, *p* = 0.233 > 0.05, respectively; the laminate lay-up method shows a significant effect (*F* = 14.89, *p* = 0.012 < 0.05).

## 4. Conclusions

In this paper, the shear strength of adhesively bonded single-lap joints were experimentally and numerically investigated. Based on the validated simulation, the effects of lap length, adhesive layer thickness, adhesive layer shape, adhesive layer overflow length, and laminate lay-up on the shear strength of adhesively bonded single-lap joints were studied. The load-displacement curves and shear strength under different parameters were compared. Moreover, the interaction between the above factors was studied to select the key factor by the Design of Experiment (DOE) method. The following conclusions were obtained:

The shear strength of single-lap joints gradually decreases with the increase in lap length. The shear strength of single-lap joints gradually decreases as the thickness of the adhesive layer increases. The shear strength gradually increases with the increase in the overflow length of the adhesive layer.

By comparing the shear strength of three overflow shapes, the shear strength of the normal-shaped adhesive layer is the largest, and the different overflow shapes have a greater effect on the shear strength of single-lap joints. According to the results, the higher the proportion of 0° lay-up, the greater the shear strength of single lap, and the maximum shear strength of the laminate with the lay-up of [0]_16s_, which was nearly 3 times larger than the one of [90]_16s_.

The lap length, adhesive layer thickness, lap overflow length, and laminate lay-up method in the interaction of each factor on tensile shear strength, the lap length, and laminate lay-up method will have a significant effect on the tensile shear strength. It will be beneficial for the selection of parameters in the design and fabrication process.

## Figures and Tables

**Table 1:** ZPNPREG^®^2551/150/37 parameters.

Mechanical Performance Parameters	Symbol	Value
Longitudinal tensile modulus	$E_{1}$	111 GPa
Transverse tensile modulus	$E_{2}$	7.3 GPa
In-plane shear modulus	$G_{12}$	3.7 GPa
Longitudinal tensile strength	$X_{t}$	1690 MPa
Longitudinal compression strength	$X_{c}$	1070 MPa
Transverse tensile strength	$Y_{t}$	35 MPa
Transverse compression strength	$Y_{c}$	134 MPa
In-plane shear strength	S	49 MPa

**Table 2:** Adhesive performance parameters.

Types of Adhesives	*K_nn_* MPa	*K_ss_* MPa	*K_tt_* MPa	*σ_n_* MPa	*σ_s_* MPa	*σ_t_* MPa	*G_nn_* N/mm	*G_ss_* N/mm	*G_tt_* N/mm
LORD^®^320/322	1586	1586	1586	30.6	30.6	30.6	0.3	1.2	1.2

**Table 3:** Experimental results of tensile shear test.

Experimental Parts	Failure Load (N)	Tensile Shear Strength (MPA)
A1	5091.36	16.25
A2	5371.08	16.93
A3	5563.76	17.82
Average value	5342.07 ± 237.53	17.00 ± 0.10

**Table 4:** Orthogonal experimental design and results.

No.	Lap Length (mm)	Adhesive Layer Thickness (mm)	Adhesive Layer Overflow Length	Laminate Lay-Up	Tensile Shear Strength (MPa)
1	12.50	0.25	0*L*	[0]_16s_	25.09
2	12.50	0.50	0.5*L*	[90]_16s_	5.91
3	12.50	0.75	*L*	[0/90]_4s_	16.73
4	12.50	1.00	0*L*	[0/±45/90]_2s_	15.27
5	25.00	0.25	0.5*L*	[0/90]_4s_	12.33
6	25.00	0.50	0*L*	[0/±45/90]_2s_	10.11
7	25.00	0.75	0*L*	[0]_16s_	12.18
8	25.00	1.00	*L*	[90]_16s_	3.19
9	37.50	0.25	*L*	[0/±45/90]_2s_	9.54
10	37.50	0.50	0*L*	[0/90]_4s_	9.33
11	37.50	0.75	0*L*	[90]_16s_	4.71
12	37.50	1.00	0.5*L*	[0]_16s_	8.84
13	50.00	0.25	0*L*	[90]_16s_	4.33
14	50.00	0.50	*L*	[0]_16s_	9.22
15	50.00	0.75	0.5*L*	[0/±45/90]_2s_	6.99
16	50.00	1.00	0*L*	[0/90]_4s_	7.39

**Table 5:** Multifactor analysis of variance results for orthogonal experiments.

Source	Sum of Squares	df	Mean Square	*F* Value	*p*
Intercept distance	1367.847	1	1367.847	326.051	0.000 **
Lap length (mm)	184.15	3	61.383	14.632	0.013 *
Adhesive layer thickness (mm)	46.295	3	15.432	3.678	0.12
Adhesive overflow length	17.984	2	8.992	2.143	0.233
Laminate lay-up	187.397	3	62.466	14.89	0.012 *
Residuals	16.781	4	4.195		

*R*^2^ = 0.963; * *p* < 0.05 ** *p* < 0.01.

## Data Availability

Not applicable.
